# Retrospective cohort of a decade of pediatric kidney transplant in a Brazilian state: Clinical profile, main complications, and outcomes

**DOI:** 10.1371/journal.pone.0323648

**Published:** 2025-05-30

**Authors:** Marina da Rocha Lordelo, Claudia Andrade Nunes, Mariana Araújo-Pereira, Beatriz Barreto-Duarte, Bruno B. Andrade

**Affiliations:** 1 Programa de Pós-Graduação em Medicina e Saúde Humana, Escola Bahiana de Medicina e Saúde Pública (EBMSP), Salvador, Bahia, Brazil; 2 Departamento de Pediatria, Faculdade de Medicina da Bahia, Universidade Federal da Bahia, Salvador, Bahia, Brazil; 3 Hospital Ana Nery, Salvador, Bahia, Brazil; 4 Complexo Hospitalar Universitário Professor Edgard Santos, Salvador, Bahia, Brazil; 5 Multinational Organization Network Sponsoring Translational and Epidemiological Research (MONSTER) Initiative, Salvador, Bahia, Brazil; 6 Instituto de Pesquisa Clínica e Translacional, Faculdade Zarns, Clariens Educação, Salvador, Brazil; 7 Laboratory of Clinical and Translational Research, Gonçalo Moniz Institute, Oswaldo Cruz Foundation, Salvador, Brazil; 8 Institute for Research in Priority Populations (IRPP), Multinational Organization Network Sponsoring Translational and Epidemiological Research (MONSTER) Initiative, Salvador, Brazil; 9 Brazilian Tuberculosis Research Network (REDE-TB), Brazil; 10 Programa de Pós-Graduação em Ciências da Saúde, Universidade Federal da Bahia, Salvador, Brazil; Medstar Georgetown Transplant Institute, UNITED STATES OF AMERICA

## Abstract

Pediatric kidney transplant is performed globally, although unevenly, with specific challenges in low-income countries with limited resources. We aimed to describe pediatric kidney transplantation in Bahia, a state located in one of the poorest regions in Brazil, and explore possible predictors of survival. This was a single-center retrospective cohort, and we included 101 pediatric kidney transplants performed between 2013 and 2022. There was no predominance of sex; the median age was 12 years old. Congenital anomalies of the kidney and urinary tract were the most common etiology of renal disease. 21 transplants were preemptive. Delayed graft function occurred in just over half of transplants. Patient survival rate was 96%, 96%, 89.1%, and 89.1% respectively at 1-year, 3-years, 5-years, and 10-years post-transplant. The overall graft survival rate was 80.2%, 76.9%, 66.8%, and 45.8% at 1-year, 3-years, 5-years, and 10-years post-transplant. Multivariate analysis of outcome predictors revealed that delayed graft function was a risk factor for graft survival in 5 years (adjusted HR 3.44 (1,18–10,05)). Pediatric kidney transplantation is a regionally feasible treatment, with good outcomes, although slightly inferior to those reported in the literature; efforts on reducing incidence in delayed graft function may improve graft survival.

## Introduction

Kidney transplant (KT) is considered the treatment of choice for kidney failure (KF) in children and teenagers, offering a chance at improved quality of life and extended survival [1,2]. This procedure is performed globally, although unevenly, because of its costs and complexity [3]. Outcomes of KT are historically improving, allowing great short-term survival of graft However, challenges with long-term outcomes persist [4–6], especially in low- and low-middle-income countries. In low resource settings, beyond medical issues, there are difficulties with family educational level, housing and sanitation conditions, food security, socioeconomic vulnerability, and health services structure [7]. In 2023, Brazil recorded a substantial transplantation activity, with more than 6000 KT procedures performed; pediatric KT accounts for about 5% of these numbers. Despite this, the rate of KT in Brazil is about 30 transplants per million people, ranking lower than in some other upper-middle-income countries [8]. Such disparities are more pronounced when examining regional differences within the country. Previous multicentric studies in our country have described pediatric kidney transplantation in Brazil [9,10]. Nevertheless, there are great inequalities of this practice: children enrolled in some regions are less likely to receive a KT, and better economic and social indicators of a region correlate with better KT numbers [11].

These regional disparities raise crucial questions about equity in healthcare access and the quality of care provided to children and adolescents with kidney failure across different Brazilian states. In this study, we aimed to describe the clinical and epidemiological characteristics of pediatric kidney transplantation and its outcomes in the state of Bahia. This is a region where the majority of the population is considered Black, and with an illiteracy rate of around 12%, just over 50% of the population have access to the sewage system, and where the nominal monthly household income per capita is around US$ 230 in the year of 2024 [12]. By focusing on this locality, the study seeks to illuminate the broader implications of regional disparities on pediatric KT outcomes in Brazil. We also performed an exploratory analysis of predictors of graft and patient survival within this cohort, providing insights that may guide future interventions and policy adjustments to improve the equity and effectiveness of kidney transplantation across diverse regions. In essence, our research question was whether KT in our location, a low-resource setting, is feasible and whether it has similar outcomes as what’s described in the literature.

## Materials and methods

### Ethical considerations

This study was approved by the Ethics Committee of Hospital Ana Nery (certificate - CCAE 75834723.2.0000.0045), and it was conducted following the Declaration of Helsinki. Due to its retrospective nature, informed consent was waived. To minimize the risk of loss of confidentiality, all participants were identified with an alphanumeric code, and all patient information entered into the database was identified only by the study code. All computer and network program entries were made using the alphanumeric code only.

### Study design and study population

This study was a single-center retrospective cohort. All children and teenagers until 18 years old who are in dialysis OR with advanced kidney disease in the state of Bahia, Brazil, are referred to the Pediatric Kidney Transplant Service at Hospital Ana Nery, a public hospital located in the city of Salvador - Bahia. The pediatric kidney transplant program began in 2009. We included all pediatric kidney transplants carried out at the hospital between January 2013 and December 2022, allowing at least 1 year and a maximum of 10 years of follow-up. We collected all clinical data until December 31, 2023 (administrative end of study). Data was accessed between December 18, 2023, and May 31, 2024. All data used in the analyses are available in S1 File.

### Data source, variables, and outcomes

Demographic and clinical parameters were captured in electronic and written medical records. In addition, the National Transplant System was consulted for some information, particularly concerning donors.

All variables of interest are listed in Supplementary Material – S1 Text, with their definition. The main ones were age, sex and race/ethnicity of recipients, weight and etiology of KF, information about treatment before KT, immunological events before transplant (as cytomegalovirus - CMV, transfusions, previous transplants, Panel Reactive Antibody-PRA), type of donor (living or deceased, age, cause of death), data about immunosuppressive therapy and about the transplantation (cold ischemia time – CIT, delayed graft function – DGF). DGF was defined as the need for dialysis within the first 7 days after KT; it was applicable only if there was not a premature graft loss (<7 days).

Our outcomes were graft function or death at hospital discharge and 1 year for all cases. If follow-up long enough, we collected these outcomes at 3-, 5- and 10-years post-transplant. Global graft survival was defined as the time between KT date and either date of graft failure (marked by retransplantation or return to dialysis) or date of death, censoring for the last date of follow-up with a functioning graft and administrative end of study. This estimate assumes that all fatal events are related to transplantation. Death-censored graft survival was defined as the time between KT date and either date of graft failure or last date of follow-up with a functioning graft, censoring for death and administrative end of study. This estimate assumes that no deaths are related to transplantation since death is handled as cases lost to follow-up. Patient survival was defined as the time from KT to death or last follow-up, censoring for administrative end of study. All the survival rates were expressed with their 95% confidence interval, as previously recommended [13]. The date of death was reported by the assistant team or family members and recorded in medical charts; causes of death were not described in detail.

Secondary outcomes of interest assessed were CMV or BK-polyomavirus infection, relevant infection requiring hospital admission, confirmed or presumed episodes of rejection and occurrence of post-transplant lymphoproliferative disease (PTLD), throughout the entire follow-up until December 2023.

### Statistical analysis

Qualitative variables were represented as frequency, while the quantitative ones were expressed by median and interquartile range (IQR). Because of frequent right censoring and the non-normality aspect of survival in transplantation, we used the Kaplan-Meier method to analyze the 1-, 3-, 5- and 10-year patient and global and death-censored graft survival, Furthermore, we selected potential candidates for predictors of unfavorable outcomes, based on literature review and clinical plausibility.. We chose to use global graft survival since death is not a common event in pediatric patients, and it should be, in theory, related to transplant. Also, we used 5-year graft survival instead of 10-year since there were already enough graft losses with an adequate number of transplants at risk after this period. The log-rank test was used to compare survival curves between groups from categorical variables, and the univariate Cox proportional hazard model was used to compare continuous variables. Variables with a P value ≤ 0.20 in the univariate analysis were selected. Some of them presented a risk of collinearity (such as those related to the size of the patient, e.g., recipient’s weight, height, and age). Thus, we tested all the selected variables for multicollinearity with the Variance Inflation Factor (VIF) so that we could improve reliability. Those with VIF > 10 were excluded, one by one, until only tolerable VIF remained. Afterward, we included the potential predictors in the multivariate analysis, also made with Cox proportional hazards regression analyses. All tests were 2-sided, with statistical significance set at a 0.05 point. All data were analyzed using the 2017 Stata software package.

## Results

### Characteristics of the study population

Our cohort comprised 101 pediatric kidney transplants performed on 95 patients, with a median follow-up time of 70 months (IQR 46–97 months). There was no predominance of sex (50,5% male), and 92% of patients were Black (n = 92) (Table 1). Median age was 12 years (IQR 9–15 years), and median weight was 28 kg (IQR 21–40.3 kg); the youngest child was 3 years old and the smallest one, 9,6 kg. Most patients (73.3%) had already received at least one blood transfusion. Retransplants were infrequent (n = 6, 5.9%). Congenital anomalies of the kidney and the urinary tract (CAKUT) were the most common etiology of kidney failure (n = 29, 28.7%), but unknown causes were also very frequent (n = 27, 26.7%). Twenty-one transplants were preemptive (20.8%); among the other 80 patients, the median time of dialysis before transplant was 16 (IQR 9–18.5) months. The majority of patients had positive serology for CMV (92.1%, n = 93); serology for Epstein-Barr virus (EBV), instead, was not known in just half of the study population (51.5%, n = 52).

**Table 1:** Demographic and clinical data from recipients of pediatric kidney transplants in Bahia, Brazil, 2013-2022.

Variables	N
**Male sex**, n (%)	50 (50.5)
**Race**, n (%)	
Black	92 (91.1)
White	8 (7.9)
Missing	1 (1)
**Age** (years), median (IQR)	12 (9-15)
**Weight** (kg), median (IQR)	28 (21-40.3)
**Height** (cm), median (IQR)	135 (120-155)
Missing, n (%)	2 (2)
**Blood transfusions before KT,** n (%)	
None	27 (26.7)
1-5	69 (68.3)
6-10 transfusions	8 (7.9)
>10 transfusions	5 (5.0)
**PRA** (%), median (IQR)	8 (0-27.1)
**Retransplant**, n (%)	6 (5.9)
**CMV serology pre-KT**, n (%)	
Positive	93 (92.1)
Negative	7 (6.9)
Unknown (missing)	1 (1)
**EBV serology pre-KT**, n (%)	
Positive	37 (36.6)
Negative	12 (11.9)
Unknown (missing)	52 (51.5)
**Cause of KF,** n (%)	
CAKUT	29 (28.7)
FSGS	17 (16.8)
Other glomerulopathies	17 (16.8)
Unknown	27 (26.7)
Other	11 (10.9)
**Treatment before KT, **n (%)	
Conservative (preemptive KT)	21 (20.8)
Peritoneal dialysis	27 (26.7)
Hemodialysis	53 (52.5)
Time in dialysis before KT^*^ (months), median (IQR)	16 (9-18.5)
N/A (preemptive transplant)	21
Time in waiting list^**^ (months), median (IQR)	6.8 (3.1-13.4)
N/A (living donor)	8

* Time between the start of dialysis, regardless of which one or if more than one, and date of transplant.

** Time between enrollment in National Transplant System and date of transplant.

IQR: interquartile range, KT: kidney transplant, PRA: panel-reactive antibody, CMV: cytomegalovirus, EBV: Epstein-Barr virus, KF: kidney failure, CAKUT: congenital anomalies of the kidney and the urinary tract, FSGS: focal and segmental glomerulosclerosis, N/A: non-applicable.

Deceased-donor KT (92.1%) was more frequent than living donor KT (n = 8, 7.9%); donors were young, and external causes of death were common (S1 Table). One patient was submitted to an *en-bloc* kidney transplant. Most transplants had cold ischemia time less than 24 hours (median time 19.4h, IQR 15.9–22.6); DGF was frequent (51.7%). Most patients (53.5%) received anti-thymocyte globulin (ATG) as induction therapy and received a combination of tacrolimus (98%), mycophenolate acid (97%), and prednisone (95%) (Table 2).

**Table 2:** Clinical data about pediatric kidney transplants in Bahia, Brazil, 2013-2022.

Variables	N
***En-bloc* transplant**, n (%)	1 (1)
**Induction therapy**, n (%)	
Basiliximab	26 (25.7)
Polyclonal ATG	52 (51.5)
Methylprednisolone	21 (20.8)
Basiliximab + polyclonal ATG	2 (2)
**Initial immunosuppression agents**, n (%)	
Tacrolimus	99 (98)
Mycophenolate acid	98 (97)
Prednisone	96 (95)
Everolimus	1 (1)
**CIT** (h), median (IQR)	19.4 (15.9-22.6)
N/A (living donor)	8
**DGF**, n (%)	
Yes	45 (49.5)
No	42 (46.1)
Missing	4 (4.4)
N/A (premature failure = graft failure < 7 days)	10

Cold ischemia time was defined as the time between donor aortic clamp and exit of cold storage. Delayed graft function was defined as the need for dialysis in the first 7 days after the kidney transplant for those with no failure in the first 7 days. Premature failure was defined as graft failure in the first seven days.

IQR: interquartile range, ATG: anti-thymocyte globulin, CIT: cold ischemia time, DGF: delayed graft function, N/A: non-applicable.

### Primary outcomes: Graft and patient survival

**Fig 1:**
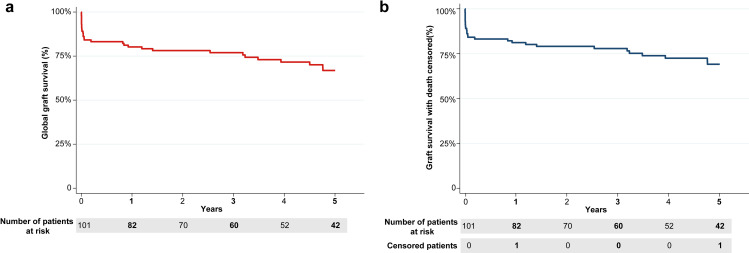
Global and death censored graft failure in 5 years. A: Global graft survival in 5 years. B: Death-censored graft survival in 5 years. Both graphs were estimated by the Kaplan-Meyer method. Death-censored graft survival treats fatal events as lost to follow-up, assuming all deaths are caused by other causes than transplantation. Patients lost to follow-up are also censored. Global graft survival represents the overall rate of success of the treatment since it treats each and every death as associated with the transplant; only loss of follow-up is censored. This metric may be more appropriate for pediatric kidney transplants.

There were 16 vascular thrombosis (15.8%); two more grafts failed in the first 90 days (primary non-function). 83 patients (82.2%) were dismissed with a functioning graft. In the first year post-transplant, there was one more graft failure and one death with a functioning graft. Thereby, 80% (16/20) of graft failure in the first year, including death as graft failure, was caused by vascular thrombosis. Global graft survival after the 1st, 3rd, 5th and 10th year was 80.2% (CI95: 71.0–86.7%), 76.9% (CI95: 67.4–84.1%), 66.8% (CI95: 55.7–75.8%) and 45.8% (CI95: 23.2–65.9%), respectively. Median graft survival time was 3440 days (or 9.4 years). In a very similar way, death-censored graft survival was 81.2% (CI95: 72.1–87.6%) at one year, 77.9% (CI95: 68.3–84.9%) at 3 years, 69.1% (CI95: 58.1–77.8%) at 5 years and 47.4% (CI95: 23.8–67.7%) at 10 years. 5-years graft survival graphs are presented in Fig 1; 10-year graft survival is presented in S3 Fig. The outcomes of all KT included throughout the follow-up are represented in S2 Fig.

The mortality rate (9.5%) was calculated based on 95 patients. Two deaths were caused by PTLD; both patients died with a functioning graft. The remaining patients (7 of 9 deaths) died after graft loss, most from infections or cardiovascular causes. Patient survival at the first, third, fifth and tenth year were 96% (CI95: 89.8–98.5%), 96.0% (CI95: 89.8–98.5%), 89.1% (CI95: 79.9–94.3%) and 89.1% (CI95: 79.9–94.3%), respectively. All the survival rates and their confidence interval are presented in Table 3.

**Table 3:** Survival rates in pediatric kidney transplantation in Bahia, Brazil, 2013-2022.

Metric	% (CI95%)	Patients at risk
**Patient survival**		101
1 year	96.0 (89.8-98.5)	97
3 years	96.0 (89.8-98.5)	76
5 years	89.1 (79.9-94.3)	57
10 years	89.1 (79.9-94.3)	7
**Global graft survival**		101
1 year	80.2 (71.0-86.7)	82
3 years	76.9 (67.4-84.1)	60
5 years	66.8 (55.7-75.8)	42
10 years	45.8 (23.2-65.9)	3
**Death-censored graft survival**		101
1 year	81.2 (72.1-87.6)	82
3 years	77.9 (68.3-84.9)	60
5 years	69.1 (58.1-77.8)	42
10 years	47.4 (23.8-67.7)	3

CI: confidence interval.

### Secondary outcomes: Infections, PTLD, and rejection

During the first year of follow-up, our incidence rate of CMV (viremia and disease) was 48.8%; this frequency fell in the subsequent years. Polyomavirus infection was less common (15.5%) but maintained a stable frequency throughout the follow-up period (13.7% between the first and third year, 15.2% between the third and fifth year). Other infections leading to hospital admission, whether viral, bacterial, or parasitic, were equally frequent (43.9% in the first year), with importance all over the ten years of follow-up (18.4% between the fifth and the tenth year). PTLD was rare (2 cases over the 10 years) but an important cause of death. Rejection (presumed or biopsy-proven) occurred in 17 patients in the first year (20.2%), in 10 patients (12.5%) between the 1^st^ and 3^rd^ year, also in 10 patients (17%) between the 3^rd^ and the 5^th^ year and in 5 (12.5%) patients between the 5^th^ and the 10^th^ year (Table 4).

**Table 4:** Incidence of clinical events of interest among pediatric kidney transplants in Bahia, Brazil, 2013-2022.

	First year	Between 1^st^ and 3^rd^ year	Between 3^rd^ and 5^th^ year	Between 5^th^ and 10^th^ year
	(84 at risk)	(80 at risk)	(59 at risk)	(38 at risk)
CMV, n (%)	41 (48.8)	3 (3.7)	0	0
Polyomavirus infection, n (%)	13 (15.5)	11 (13.7)	9 (15.2)	3 (7.9)
Relevant infections, n (%)	36 (43.9)	18 (22.5)	11 (18.6)	7 (18.4)
Rejection (presumed or confirmed), n (%)	17 (20.2)	10 (12.5)	10 (17)	5 (13.1)
PTLD, n (%)	1 (1.2)	0	1 (1.7)	0

The Number of patients at risk refers to functioning grafts at the beginning of the period. CMV infection was defined as positive PCR-CMV OR positive antigenemia OR histological changes suggestive of CMV infection THAT motivated antiviral treatment (with ganciclovir or valganciclovir) OR modification of immunosuppression. Polyomavirus infection was defined as positive urine BKPyV-DNAuria OR plasma BKPyV-DNAemia OR presence of decoy cells OR histological changes suggestive of BKPyV infection THAT motivated modification of immunosuppression. Relevant infection: Viral (except for CMV or BKPyV), bacterial, fungal, or parasitic infection, presumed or confirmed, leading to hospital admission. Rejection: whether presumed (physician’s decision to initiate specific anti-rejection therapy) OR confirmed (biopsy-proven) rejection episode.

CMV: cytomegalovirus, PTLD: post-transplant lymphoproliferative disease.

### Predictors of graft survival and patient mortality

We analyzed several potential predictors of global graft survival. In a univariate analysis, age, weight and height of the recipient, donor final creatinine and weighting less than 15 kg, DGF, Human Development Index (HDI) of the city of residence (as a surrogate of socioeconomic status), CAKUT and FSGS as etiology of kidney failure, were correlated with graft survival. The age and height of the recipient were excluded from the multicollinearity test. In multivariate analysis, only DGF remained as a risk factor for global graft survival (HR 3.44, with a CI95: 1.18–10.05, P = 0.02) (Table 5 and S4 Fig). The recipient’s weight was marginally correlated with better outcomes.

**Table 5:** Predictors of graft survival in pediatric kidney transplantation in Bahia, Brazil, 2013-2022.

	Univariate analysis		Multivariate analysis	
Variables	HR (CI95%)	p-value	HR (CI95%)	p-value
Recipient age	0.90 (0.83-0.98)	0.02	–	
Recipient weight	0.97 (0.94-1.00)	0.05	0.95 (0.91-1.00)	0.053
Recipient height	0.98 (0.97-1.00)	0.04	–	
Female recipient	–	0.95	–	
Black race	–	0.67	–	
CAKUT	2.00 (0.96-4.15)	0.06	1.2 (0.40-3.73)	0.73
FSGS	0.33 (0.78-1.37)	0.11	0.67 (0.13-3.37)	0.63
Blood transfusions	–	0.46	–	
PRA	–	0.79	–	
More than 1 modality of dialysis	–	0.71	–	
Peritoneal dialysis pre-KT	–	0.23	–	
Preemptive transplant	–	0.34	–	
Waiting time	–	0.36	–	
Dialysis time before KT	–	0.39	–	
Retransplant	–	0.98	–	
Living donor	–	0.70	–	
Donor age	–	0.63	–	
Donor less than 15 kg	2.20 (0.66-7.24)	0.19	0.36 (0.04-3.28)	0.36
Donor final creatinine	0.56 (0.26-1.20)	0.13	0.69 (0.26-1.86)	0.47
DGF	3.23 (1.14-9.15)	0.02	3.44 (1.18-10.05)	**0.02**
CIT (minutes)	–	0.40	–	
Induction with ATG	–	0.53	–	
HDI of the city of residence	0.05 (0.00-4.79)	0.20	0.01 (0.00-10.1)	0.20

Potential predictors were selected by literature review. The log-rank test was used to compare survival curves between groups from categorical variables, and the univariate Cox proportional hazard model was used to compare continuous variables. Variables with a P value ≤ 0.20 in the univariate analysis were selected and tested for multicollinearity; recipient age and height were then excluded. The remained variables were included in the multivariate analysis, also made with Cox proportional hazards regression analyses. Statistical significance (p < 0.05) is highlighted in bold.

HR: hazard ratio, CI: confidence interval, KT: kidney transplant, CAKUT: congenital anomalies of the kidney and the urinary tract, PRA: panel-reactive antibody, FSGS: focal and segmental glomerulosclerosis, KF: kidney failure, DGF: delayed graft function, CIT: cold ischemia time, ATG: antithymocyte globulin, HDI: Human Development Index.

We also analyzed patient survival and its association with the same variables. In a univariate analysis, the age, weight, and height of the recipient were considered protective of patient survival; on the other hand, peritoneal dialysis pre-KT, more than one modality of dialysis, presence of DGF, and living donor were considered risk factors for worse patient survival. The age and height of the recipient were also excluded in the multicollinearity test. In multivariate analysis, we found no predictors of mortality in 10 years (S2 Table).

## Discussion

The present study demonstrates that patient and graft survival rates at a single Brazilian center were comparable to those reported by most transplantation cohorts. Clinical and demographic characteristics were generally similar; however, some differences were noted, including a higher proportion of Black recipients, a greater prevalence of kidney failure (KF) with unknown etiology, a longer waiting times for transplantation and a longer period on dialysis compared to most previously published studies [10,14–18]_._ Furthermore, our exploratory analysis identified an adverse association between DGF and graft survival. No significant predictors of patient survival were found. The predominance of black patients is in line with demographic data from our state in Brazil [12]. This is quite different from other studies from Brazil [12,18] and other countries [5,17,19,20]. Of note, the collaborative Brazilian registry did not mention the race of the patients [10]. Race has been identified as a predictor of transplant outcomes since a huge number of studies have described that black patients have a higher risk of graft failure, either among adults or pediatric patients [17,21–23]. The black race can be considered a marker of a combination of social determinants of health capable of justifying worse outcomes, as low socioeconomic status, poor access to better quality health services, food, and job insecurity. Previous literature data have already shown that black patients have worse graft survival even after adjusting for various sociodemographic and even clinical characteristics [24]. Hence, immunological, biological, and genetic factors that are not yet fully understood may also account for poor outcomes in Black patients. Over the last decade, for instance, the discovery of a higher prevalence of APOL1 risk variants in individuals with recent African ancestry has illuminated some questions about this racial disparity [25]. Our study was not able to assess variables related to socioeconomic status individually and did not aim to evaluate APOL1 gene expression. Nevertheless, the great majority of black patients in our cohort may be a possible, albeit partial, explanation for our finding of slightly worse graft survival compared to other published cohorts.

Our study population has a similar etiology of KF and sex distribution of previously described transplanted children[10,17]. The frequency of patients with unknown etiology of renal failure, however, was much higher than described in other contexts [17,26]; this is generally common in low-resource settings, when early and primary diagnosis of chronic kidney disease can be quite challenging. This explanation is endorsed by a previous study that revealed a higher rate of unknown primary diagnosis in the poorest regions of Brazil (up to 46.3%) and a minimum time between primary chronic kidney disease diagnosis and initiation of renal replacement therapy in these regions (less than 1 month) [27].

We have a substantial number of preemptive transplants, greater than some other Brazilian centers[10,14,15]. Time in dialysis before KT is higher than our waiting time, revealing that our children and adolescents remain on dialysis for some time before they are enrolled in the National Transplant System or even without being referred to a transplantation center for preparation. In agreement with Brazilian cohorts [10,15,28], deceased donor KT was far more frequent than living donor KT in our population. In other countries, the rate of transplants with a living donor exceeds that with a deceased donor, to a greater or lesser extent [16,17,19]. This divergence may reflect discrepancies between living organ donation policies between countries, in combination with differences in the structure of organ procurement systems from deceased donors. In Brazil, there are legal restrictions on unrelated kidney donation, and transplantation with a living donor accounted for less than 15% of all kidney transplant activity in the country in 2023 [8].

Furthermore, DGF was very prevalent in our study population, even with CIT shorter than 24 hours, differently from what has been reported in other pediatric Brazilian cohorts [10,15], but following rates presented in a multicenter study of adults in our country [29]. Poor donor maintenance, recipient dialysis adequacy, or immunological factors (as HLA mismatches or preformed HLA antibodies) may account for this high prevalence; unfortunately, these features were not captured in our study.

Use of ATG was very frequent in our population, and this may be related to the unavailability of basiliximab in our center since 2017. This augmented use of induction with lymphocyte-depleting agents may be at least in part responsible for our high incidence of CMV infection (whether viremia or disease). On the other hand, we had a relatively high frequency of patients with presumed or confirmed rejection, similar to what’s described in the North American registry [17], and higher than reported in the Brazilian study [10].

Patient survival was slightly inferior to what has been described in pediatric Brazilian studies [10,14]; however, it was within what would be expected for data relating specifically to South America in a recently published metanalysis [30]. As for graft survival, we have worse outcomes mainly at 1-year post-transplant, at least partly due to our high rates of vascular thrombosis. Overall graft survival at 3, 5, and 10 years was also lower than that described in the meta-analysis, both for pooled global data and specific to South America [30].

Our state is one of the poorest in the country, with low per capita income and low educational levels [12]. Besides, our center has been performing pediatric KT for about 15 years, and probably our team expertise is still evolving. Innovations related to the diagnosis and treatment of some complications have been available for less time than in other specialized centers in Brazil or around the world. These factors may also contribute to our outcomes.

In our exploratory analysis of predictors of graft survival, we found that DGF had inferior outcomes; this is in concordance with what’s now known for the transplantation community, both for adults and children [17,29,31,32]. In our cohort, DGF was strongly correlated with worst graft survivals. Regarding to the predictors of patient survival, we found no predictors of mortality, possibly because of our small sample size and a small number of deaths. To our knowledge, there are few studies that correlate predictors of patient survival rate in pediatric population. NAPRTCS reports better patient survival with living donor kidney transplants and with recipients > 2 years old.

This study has some limitations. The retrospective nature may lead tosome inconsistent information throughout all the follow-up. Particularly, data about race may have not been self-declared in all patients. Also, our study population only included patients attending in our public health system. We had little missing data. However, due to the frequent unavailability of some data in the medical records, some variables were not included in the study, as number of HLA compatibilities or social and economic features, In addition, we had a small number of deaths; thus, our analysis of predictors of patient survival must be confirmed in a larger population. These findings may have some influence of race or other social and clinical characteristics, not captured in this study, such as biochemical data or dialysis adequacy before KT.

Despite the limitations, this is the first report from a peripheral Brazilian region, showing that kidney transplant is still a good and feasible option for KF treatment regionally, allowing the survival of patients with this complex and severe disease. Based on our findings, we reinforce the need for early referral to transplantation centers and special attention to small children. We urge for better care of pediatric patients with chronic kidney disease in our location and in other low resource settings, with proper elucidation of primary disease, early diagnosis and expeditious preparation of the potential recipient of kidney transplant. Improving dialysis adequacy, encouraging preemptive transplantation, and optimizing potential donor maintenance can also mitigate the occurrence of DGF, allowing better outcomes. Future research is demanded for clarifying the impact of other predictors in graft and patient survival in pediatric kidney transplants. Remarkably, multicentric studies may be more promising, given the possibility of bigger sample sizes, once pediatric chronic kidney disease is a rare disease.

## Supporting information

S1 File Database with raw data. (XLS)

S1 Text Variables of interest AND Definitions. (DOCX)

S1 Table Donors of pediatric kidney transplant. (DOCX)

S2 Table Predictors of patient survival in pediatric kidney transplantation. (DOCX)

S3 Table Detailed univariate analysis. (DOCX)

S1 Fig Flow diagram of pediatric kidney transplants included and excluded from the study. (DOCX)

S2 Fig Flow diagram of follow-up and outcomes of pediatric kidney transplants included in the study. (DOCX)

S3 Fig Graft survival graphs in 10 years. (DOCX)

S4 Fig Impact of DGF in global graft survival in 5 years. (DOCX)

S5 Fig Patient survival graph in 10 years. (DOCX)
