# Supplementary material for: Retrospective cohort of a decade of pediatric kidney transplant in a Brazilian state: Clinical profile, main complications, and outcomes
Source: PLoS One. 2025 May 30;20(5):e0323648. doi: 10.1371/journal.pone.0323648 (PMC12124757; doi:10.1371/journal.pone.0323648)
Supplement: S1 Text — (DOCX) [file pone.0323648.s002.docx]

**Variables of interest AND Definitions**

Social and demographic variables: biological sex, age at KT, race (preferably self-declared in Social Service admission OR as registered at hospital enrollment), city of birth, city of residence and its Human Development Index (HDI)

Clinical variables:

Anthropometric measures: weight (kilograms) and height (centimeters) immediately before KT (at hospital admission OR at appointment prior to KT)

About kidney disease: etiology (detailed form AND categorized as follows: CAKUT - congenital anomalies of the kidney and the urinary tract, FSGS - focal and segmental glomerulosclerosis, non-FSGS glomerulopathies, unknown or other), previous treatment (hemodialysis, peritoneal dialysis OR conservative – preemptive transplant), waiting time (defined as time between enrollment in National Transplant System and date of transplant), dialysis time for non-preemptive transplants (Time between the start of dialysis, regardless of which one or if more than one, and date of transplant), other comorbidities.

Immunological and serological status prior to KT: ABO blood type, PRA: panel-reactive antibody (class I and 2 OR greater percentage between the two of them), serology for HIV, hepatitis B and C, CMV IgG, EBV IgG, number of blood transfusions reported before KT (categorized as none, 1 to 5, 6 to 10 or more than 10).

Variables about the donor: deceased or related living donor, biological sex, weight (in kilograms) and height (in centimeters) – as registered for National Transplant System if deceased donor OR at hospital admission or at appointment prior to KT if living donor, serology status for HIV, hepatitis B and C, CMV and EBV IgG, comorbidities. For deceased donors, cause of death and final creatinine.

Variables about the transplant: date of transplant, first transplant or retransplant, single KT or *en-bloc* KT, cold ischemia time (time between donor aortic clamp and exit of cold storage), occurrence of delayed graft function (dialysis in the first 7 days after the KT) in those KT with no failure in the first 7 days.

Variables about immunosuppressive therapy: induction therapy (anti-thymocyte globulin, anti-interleukin-2 receptor monoclonal antibody, methylprednisolone or other), initial oral immunosuppression (tacrolimus or cyclosporine, mycophenolate mofetil or sodium, azathioprine, prednisone, sirolimus), change of oral immunosuppressive therapy during first hospital stay.

Primary outcomes: graft function or death.

Censors: date of death, date of graft failure (marked by retransplantation or return to dialysis), date of last appointment with functioning graft (lost of follow-up) OR end of study (December 31^st^, 2023)

Primary non-function: graft failure in the 90 first days

Premature failure: graft failure in the first 7 days

Secondary outcomes

CMV infection: positive PCR-CMV OR positive antigenemia OR histological changes suggestive of CMV infection THAT motivated antiviral treatment (with ganciclovir or valganciclovir) OR modification of immunosuppression.

Polyomavirus infection: positive urine BKPyV-DNAuria OR plasma BKPyV-DNAemia OR presence of decoy cells OR histological changes suggestive of BKPyV infection THAT motivated modification of immunosuppression.

Rejection: whether presumed (physician’s decision to initiate specific anti-rejection therapy) OR confirmed (biopsy proven).

Relevant infection: viral (except for CMV or BKPyV), bacterial, fungal or parasitic infection, presumed or confirmed, leading to hospital admission

Modification of immunosuppressive therapy
