# Supplementary material for: Retrospective cohort of a decade of pediatric kidney transplant in a Brazilian state: Clinical profile, main complications, and outcomes
Source: PLoS One. 2025 May 30;20(5):e0323648. doi: 10.1371/journal.pone.0323648 (PMC12124757; doi:10.1371/journal.pone.0323648)
Supplement: S1 Table — (DOCX) [file pone.0323648.s003.docx]

**S1 Table. Donors of pediatric kidney transplant.**

| **Variables** | **N** |
| --- | --- |
| **Donor type**, n (%) |  |
| Deceased (DD) | 93 (92.1) |
| Living (LD) | 8 (7.9) |
| **Donor age** (years), median (IQR) | 17 (12-31) |
| LD, median (IIQ) | 34 (31-42) |
| DD, median (IIQ) | 17 (11-25) |
| **Donor sex**, n (%) |  |
| Male | 62 (61.4) |
| Female | 39 (38.6) |
| **Donor cause of death**, n (%) |  |
| traumatic brain injury / firearm injury / drowning | 58 (62.4) |
| stroke | 12 (12.9) |
| other causes | 23 (24.7) |
| N/A (living donor) | 8 |

Abbreviations: DD: deceased donor, LD: living donor, IQR: interquartile range, ATG: anti-thymocyte globulin, CIT: cold ischemia time, DGF: delayed graft function, N/A: non-applicable
