# Supplementary material for: Retrospective cohort of a decade of pediatric kidney transplant in a Brazilian state: Clinical profile, main complications, and outcomes
Source: PLoS One. 2025 May 30;20(5):e0323648. doi: 10.1371/journal.pone.0323648 (PMC12124757; doi:10.1371/journal.pone.0323648)
Supplement: S2 Table — (DOCX) [file pone.0323648.s004.docx]

**S2 Table. Predictors of patient survival in pediatric kidney transplantation.**

|  | **Univariate analysis** | | **Multivariate analysis** | |
| --- | --- | --- | --- | --- |
| **Variables** | **HR (CI95%)** | **p-value** | **HR (CI95%)** | **p-value** |
| Recipient age | 0.79 (0.66-0.94) | 0.01 | - | |
| Recipient weight | 0.95 (0.89-1.01) | 0.11 | **0.90 (0.81-1.00)** | **0.04** |
| Recipient height | 0.97 (0.94-1.00) | 0.03 | - | |
| Female recipient | - | 0.58 | - | |
| Black race | - | 0.70 | - | |
| CAKUT | - | 0.67 | - | |
| FSGS | - | 0.73 | - | |
| Blood transfusions | - | 0.67 | - | |
| PRA | - | 0.43 | - | |
| Previous treatment of KF | - | 0.18 | - | |
| Peritoneal dialysis pre-KT | 9.84 (2.05-47.39) | 0.01 | **7.8 (1.53-39.6)** | **0.01** |
| Preemptive transplant | - | 0.55 | - | |
| Waiting time | - | 0.38 | - | |
| Dialysis time before KT | - | 0.80 | - | |
| Retransplant | - | 0.42 | - | |
| Living donor | 3.02 (0.63-14.56) | 0.15 | 0.67 (0.75-6.02) | 0.72 |
| Donor age | - | 0.85 | - | |
| Donor less than 15kg | - | 0.52 | - | |
| Donor final creatinine | - | 0.44 | - | |
| DGF | 3.43 (0.69-17.10) | 0.11 | 2.75 (0.55-13.88) | 0.20 |
| CIT (minutes) | - | 0.39 | - | |
| Induction with ATG | - | 0.63 | - | |
| HDI of the city of residence | - | 0.35 | - | |

Abbreviations: HR: hazard ratio, CI: confidence interval, KT: kidney transplant, CAKUT: congenital anomalies of the kidney and the urinary tract, PRA: panel-reactive antibody, FSGS: focal and segmental glomerulosclerosis, ESRD: end-stage renal disease, DGF: delayed graft function, CIT: cold ischemia time, ATG: antithymocyte globulin, HDI: Human Development Index

Table notes: Cold ischemia time was defined as time between donor aortic clamp and exit of cold storage, delayed graft function was defined as the need for dialysis in the first 7 days after the kidney transplant for those with no failure in the first 7 days. Dialysis time before KT was defined as time between the start of dialysis, regardless of which one or if more than one, and date of transplant. Waiting time was defined as time between enrollment in National Transplant System and date of transplant.

Potential predictors were selected by literature review. The log-rank test was used to compare survival curves between groups from categorical variables and the univariate Cox proportional hazard model was used to compare continuous variables. Variables with a P value ≤ 0.20 in the univariate analysis were selected and tested for multicollinearity; recipient age and height were then excluded. The remained variables were included in the multivariate analysis, also made with Cox proportional hazards regression analyses. Statistical significance (p < 0.05) is highlighted in bold.
