# Supplementary material for: Retrospective cohort of a decade of pediatric kidney transplant in a Brazilian state: Clinical profile, main complications, and outcomes
Source: PLoS One. 2025 May 30;20(5):e0323648. doi: 10.1371/journal.pone.0323648 (PMC12124757; doi:10.1371/journal.pone.0323648)
Supplement: S3 Table — (DOCX) [file pone.0323648.s005.docx]

**S3 Table. Predictors of graft survival in pediatric kidney transplantation in Bahia, Brazil, 2013-2022, with detailed univariate analysis.**

|  | **Univariate analysis** | |
| --- | --- | --- |
| **Variables** | **HR (CI95%)** | **p-value** |
| Recipient age | 0.90 (0.83-0.98) | **0.02** |
| Recipient weight | 0.97 (0.94-1.00) | **0.05** |
| Recipient height | 0.98 (0.97-1.00) | **0.04** |
| Female recipient | 1.02 (0.50-2.10) | 0.95 |
| Black race | 0.78 (0.24-2.57) | 0.67 |
| CAKUT | 2.00 (0.96-4.15) | **0.06** |
| FSGS | 0.33 (0.78-1.37) | **0.11** |
| Blood transfusions | 0.78 (0.46-1.31) | 0.46 |
| PRA | 1.00 (0.99-1.02) | 0.79 |
| More than 1 modality of dialysis | 0.83 (0.31-2.21) | 0.71 |
| Peritoneal dialysis pre-KT | 1.56 (0.74-3.27) | 0.23 |
| Preemptive transplant | 0.61 (0.21-1.74) | 0.34 |
| Waiting time | 0.98 (0.93-1.03) | 0.36 |
| Dialysis time before KT | 0.99 (0.96-1.02) | 0.39 |
| Retransplant | 1.01 (0.24-4.25) | 0.98 |
| Living donor | 1.26 (0.38-4.16) | 0.70 |
| Donor age | 0.99 (0.97-1.02) | 0.63 |
| Donor less than 15kg | 2.20 (0.66-7.24) | **0.19** |
| Donor final creatinine | 0.56 (0.26-1.20) | **0.13** |
| DGF | 3.23 (1.14-9.15) | **0.02** |
| CIT (minutes) | 1.00 (1.00-1.00) | 0.40 |
| Induction with ATG | 0.79 (0.39-1.63) | 0.53 |
| HDI of the city of residence | 0.05 (0.00-4.79) | **0.20** |

Abbreviations: HR: hazard ratio, CI: confidence interval, KT: kidney transplant, CAKUT: congenital anomalies of the kidney and the urinary tract, PRA: panel-reactive antibody, FSGS: focal and segmental glomerulosclerosis, KF: kidney failure, DGF: delayed graft function, CIT: cold ischemia time, ATG: antithymocyte globulin, HDI: Human Development Index.

Table note: Potential predictors were selected by literature review. The log-rank test was used to compare survival curves between groups from categorical variables and the univariate Cox proportional hazard model was used to compare continuous variables. Variables with a P value ≤ 0.20 in the univariate analysis are highlighted in bold; they were selected and tested for multicollinearity; recipient age and height were then excluded. The remained variables were included in the multivariate analysis. Results of the multivariate analysis are shown in Table 4.
