# Supplementary material for: Retrospective cohort of a decade of pediatric kidney transplant in a Brazilian state: Clinical profile, main complications, and outcomes
Source: PLoS One. 2025 May 30;20(5):e0323648. doi: 10.1371/journal.pone.0323648 (PMC12124757; doi:10.1371/journal.pone.0323648)
Supplement: S1 Fig — (DOCX) [file pone.0323648.s006.docx]

**Flow diagram of pediatric kidney transplants included and excluded from the study.**

**
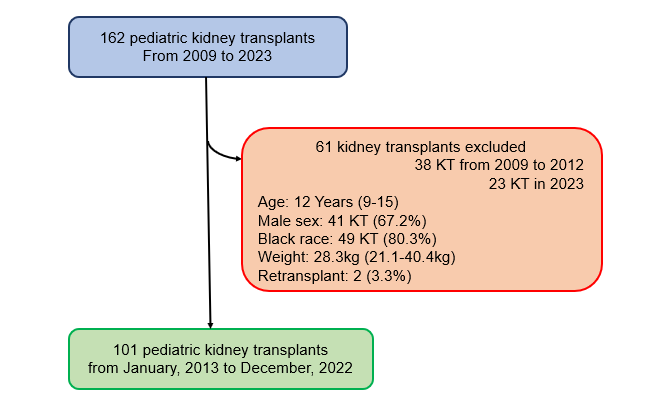
**Note: KT: kidney transplant
