# Supplementary material for: Retrospective cohort of a decade of pediatric kidney transplant in a Brazilian state: Clinical profile, main complications, and outcomes
Source: PLoS One. 2025 May 30;20(5):e0323648. doi: 10.1371/journal.pone.0323648 (PMC12124757; doi:10.1371/journal.pone.0323648)
Supplement: S2 Fig — (DOCX) [file pone.0323648.s007.docx]

**S2 Figure. Flow diagram of follow-up and outcomes of pediatric kidney transplants included in the study.**


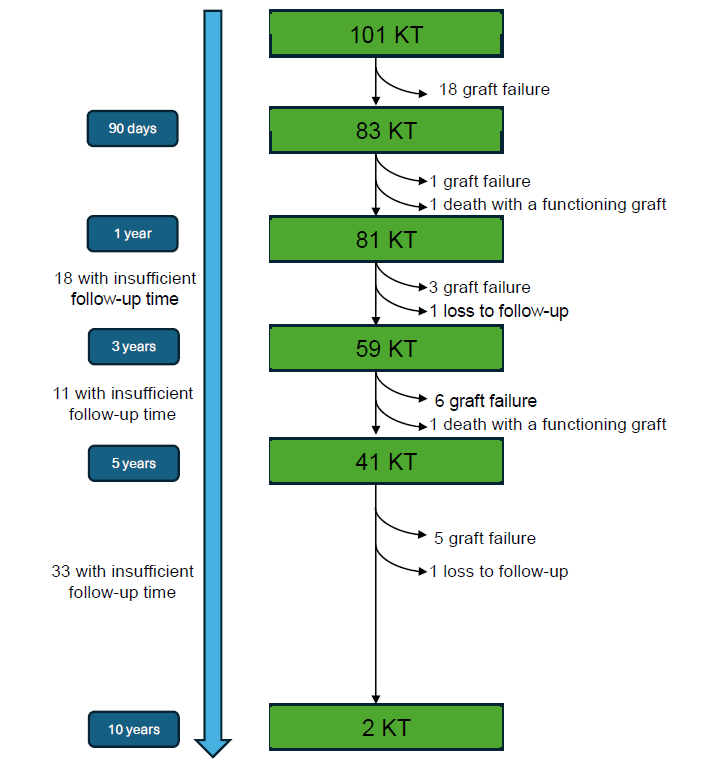
 Note: KT: kidney transplant.

This flow diagram shows the follow-up of all KTs included in the study. There were 9 deaths during the reported period. In addition to the 2 fatal events represented in the figure above, 3 children died shortly after graft loss, while still under the care of the transplant team. 4 other patients died some time after graft loss, while under the care of the dialysis team.
