# Supplementary material for: Retrospective cohort of a decade of pediatric kidney transplant in a Brazilian state: Clinical profile, main complications, and outcomes
Source: PLoS One. 2025 May 30;20(5):e0323648. doi: 10.1371/journal.pone.0323648 (PMC12124757; doi:10.1371/journal.pone.0323648)
Supplement: S3 Fig — (DOCX) [file pone.0323648.s008.docx]

**S3 Figure. Graft survival graphs in 10 years.**


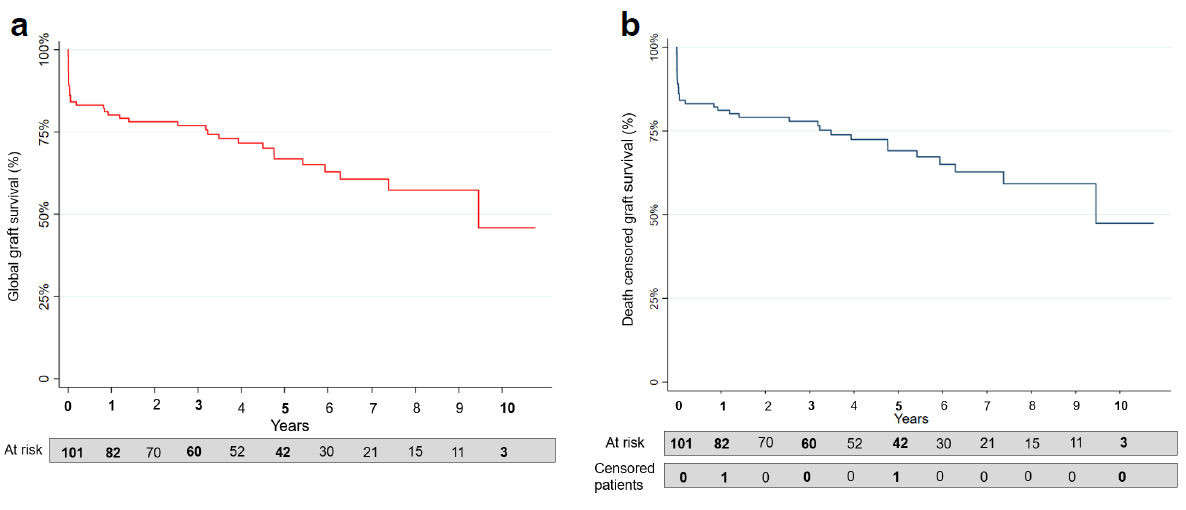
Notes: A: Global graft survival. B: Death-censored graft survival. Both graphs were estimated by Kaplan-Meyer method. Death-censored graft survival treats fatal events as lost of follow-up, assuming all deaths are caused by other causes than transplantation. Patients lost of follow-up are also censored. Global graft survival represents the overall rate of success of the treatment, since it treats each and every death as associated with the transplant; only loss of follow-up is censored. This metric may be more appropriate for pediatric kidney transplants.
