# Supplementary figures and images for: Retrospective cohort of a decade of pediatric kidney transplant in a Brazilian state: Clinical profile, main complications, and outcomes
Source: PLoS One. 2025 May 30;20(5):e0323648. doi: 10.1371/journal.pone.0323648 (PMC12124757; doi:10.1371/journal.pone.0323648)

**S4 Figure. Impact of DGF in 5 years global graft survival.**


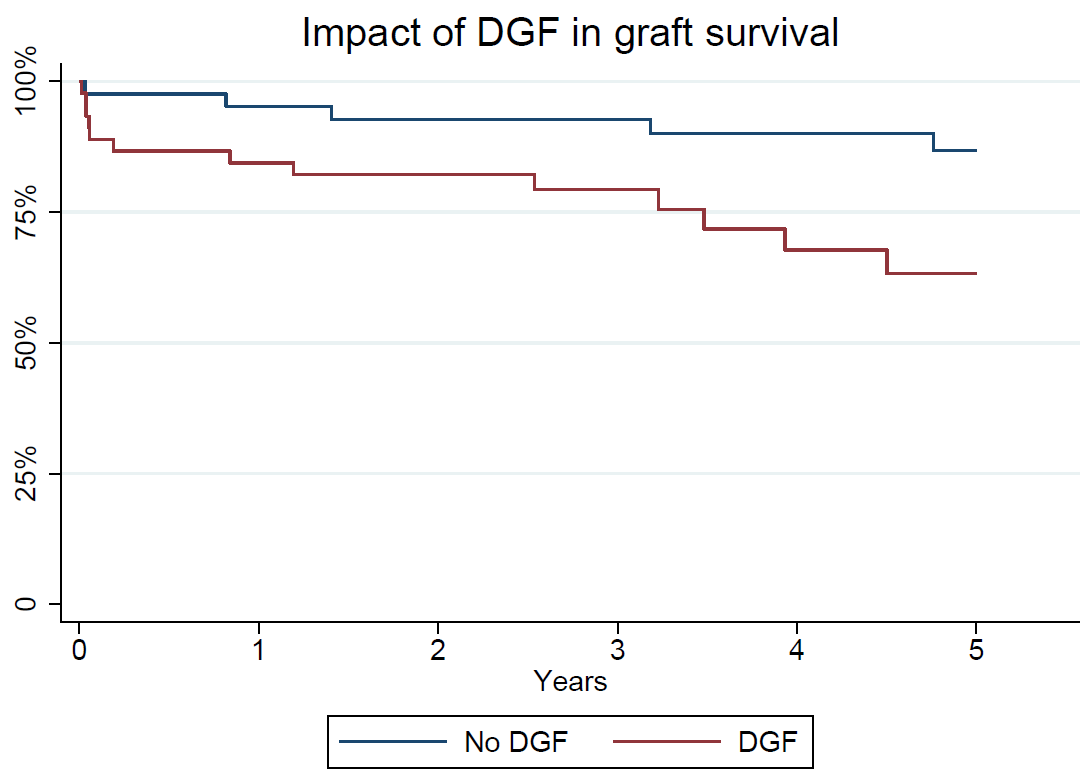


Notes: DGF: delayed graft function.

Supplement: S4 Fig — (DOCX) [file pone.0323648.s009.docx]
