# Supplementary material for: Retrospective cohort of a decade of pediatric kidney transplant in a Brazilian state: Clinical profile, main complications, and outcomes
Source: PLoS One. 2025 May 30;20(5):e0323648. doi: 10.1371/journal.pone.0323648 (PMC12124757; doi:10.1371/journal.pone.0323648)
Supplement: S5 Fig — (DOCX) [file pone.0323648.s010.docx]

**S5 Figure. Patient survival graph in 10 years.**


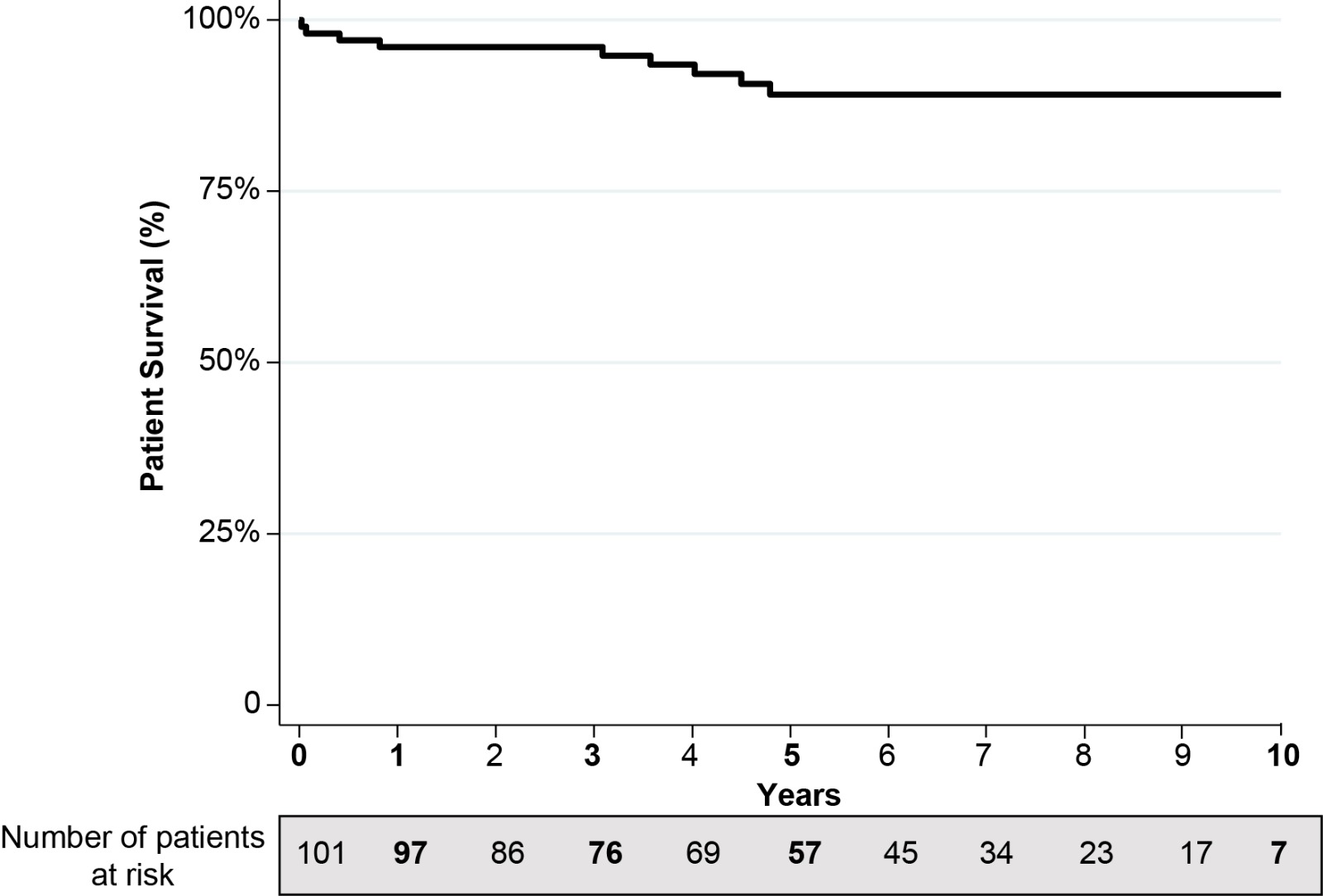


Patient survival graph was estimated by Kaplan-Meyer method. It points out the probability of death after one patient has received a kidney transplant, in a given period of time. All fatal events are counted, even those occurring long after graft loss.
